# Rocuronium blockade reversal with sugammadex vs. neostigmine: randomized study in Chinese and Caucasian subjects

**DOI:** 10.1186/1471-2253-14-53

**Published:** 2014-07-12

**Authors:** Xinmin Wu, Helle Oerding, Jin Liu, Bernard Vanacker, Shanglong Yao, Vegard Dahl, Lize Xiong, Casper Claudius, Yun Yue, Yuguang Huang, Esther Abels, Henk Rietbergen, Tiffany Woo

**Affiliations:** 1 Peking University First Hospital, No 8 Xishiku St, Beijing 100034, China; 2 Department of Anaesthesiology, Vejle Hospital, Vejle, Denmark; 3 West China Hospital, Sichuan University, Chengdu, China; 4 Department of Anesthesiology, University Hospitals Leuven, Leuven, Belgium; 5 Union Hospital, Tongji Medical College, Wuhan, China; 6 Anaesthesia and Intensive Care, Asker and Baerum Hospital, Oslo, Norway; 7 Department of Anesthesiology, Xijing Hospital, The Fourth Military Medical University, Xi’an, China; 8 Department of Anaesthesia, Hillerød Hospital, Hillerød, Denmark; 9 Beijing Chaoyang Hospital, Beijing, China; 10 Peking Union Medical College Hospital, Beijing, China; 11 MSD, Oss, The Netherlands; 12 Merck Sharp & Dohme Corp, Whitehouse Station, NJ, USA

**Keywords:** Sugammadex, Rocuronium, Neostigmine, Neuromuscular blockade, Chinese, Caucasian

## Abstract

**Background:**

This study compared efficacy and safety of the selective relaxant binding agent sugammadex (2 mg/kg) with neostigmine (50 μg/kg) for neuromuscular blockade (NMB) reversal in Chinese and Caucasian subjects.

**Methods:**

This was a randomized, active-controlled, multicenter, safety-assessor-blinded study (NCT00825812) in American Society of Anesthesiologists Class 1-3 subjects undergoing surgery with propofol anesthesia. Rocuronium 0.6 mg/kg was administered for endotracheal intubation, with 0.1–0.2 mg/kg maintenance doses given as required. NMB was monitored using TOF-Watch^®^ SX. At second twitch reappearance, after last rocuronium dose, subjects received sugammadex 2 mg/kg or neostigmine 50 μg/kg plus atropine 10–20 μg/kg, according to randomization. Primary efficacy variable was time from sugammadex/neostigmine to recovery of the train-of-four (TOF) ratio to 0.9.

**Results:**

Overall, 230 Chinese subjects (sugammadex, n = 119, neostigmine, n = 111); and 59 Caucasian subjects (sugammadex, n = 29, neostigmine, n = 30) had evaluable data. Geometric mean (95% CI) time to recovery to TOF ratio 0.9 was 1.6 (1.5–1.7) min with sugammadex vs 9.1 (8.0–10.3) min with neostigmine in Chinese subjects. Corresponding times for Caucasian subjects were 1.4 (1.3–1.5) min and 6.7 (5.5–8.0) min, respectively. Sugammadex 2 mg/kg was generally well tolerated, with no serious adverse events reported. There was no residual NMB or recurrence of NMB.

**Conclusion:**

Both Chinese and Caucasian subjects recovered from NMB significantly faster after sugammadex 2 mg/kg vs neostigmine 50 μg/kg, with a ~5.7 times (*p* < 0.0001) faster recovery with sugammadex vs neostigmine in Chinese subjects. Sugammadex was generally well tolerated.

**Trial registration:**

ClinicalTrials.gov Identifier: NCT00825812.

## Background

Neuromuscular blockade (NMB) is often used during surgery to facilitate tracheal intubation and to improve surgical conditions. Acetylcholinesterase inhibitors such as neostigmine are commonly administered to reverse NMB at the end of surgery and to reduce the risk of residual paralysis and associated adverse respiratory events [1,2]. However, these agents may provide slow and unpredictable recovery [3], and are associated with several unwanted side-effects, both alone and in combination with anticholinergic agents [4,5].

The selective relaxant-binding agent sugammadex (Bridion®, MSD, Oss, The Netherlands) has been shown to rapidly and completely reverse the effects of the NMB agents rocuronium [3,6] and vecuronium [7]. Furthermore, sugammadex is equally effective for reversal of rocuronium-induced NMB under both propofol and sevoflurane maintenance anesthesia [6]. Sugammadex is marketed for reversal of rocuronium- and vecuronium-induced NMB in over 40 countries worldwide.

Data on the efficacy and safety of sugammadex in Chinese subjects are required. The primary objective of the present study was to investigate efficacy and safety of sugammadex 2 mg/kg compared with neostigmine 50 μg/kg plus atropine 10–20 μg/kg for reversal of moderate rocuronium-induced NMB in Chinese and Caucasian subjects. Key secondary objectives were to show faster recovery from rocuronium-induced NMB with sugammadex vs neostigmine in Caucasian subjects and to demonstrate equivalence in recovery times between Chinese and Caucasian subjects.

## Methods

### Study subjects and study design

This was a randomized, parallel-group, multicenter, safety-assessor-blinded study (http://www.clinicaltrials.gov; NCT00825812; protocol P05768), conducted from February to September, 2010. The study was conducted at six sites in China and four sites in Europe (two sites in Denmark and one site each in Belgium and Norway). The study was conducted in accordance with principles of Good Clinical Practice and was approved by the appropriate Institutional Review Board (IRB)/Independent Ethics Committee at each study site. For the University Hospitals Leuven, IRB approval was provided by UZ Gasthuisberg Central IRB/EC, Leuven, Belgium. For the Vejle Hospital, IRB approval was provided by The Ethical Committee of Science for the Syddanmark Region, Vejle, Denmark. For the Asker and Baerum Hospital, IRB approval was provided by the Regional Committee for Medical Research Ethics for South-East Norway (B), Oslo, Norway. For the Peking Union Medical College Hospital, IRB approval was provided by the Peking Union Medical College Hospital IRB/EC, Beijing, China. For the Beijing Chaoyang Hospital, IRB approval was provided by Beijing Chaoyang Hospital IRB/EC, Beijing, China. For the Peking University First Hospital, IRB approval was provided by Peking University First Hospital IRB/EC, Beijing, China. For the West China Hospital, IRB approval was provided by West China Hospital IRB/EC, Sichuan, China. For the Xijing Hospital, IRB approval was provided by Xijing Hospital affiliated to Fourth Military Medical University IRB/EC, Xi’an, China. For the Union Hospital, IRB approval was provided by Union Hospital Tongj Medical College Huazhong University of Science IRB/EC, Hibei Province, China. For the Hillerød Hospital, IRB approval was provided by The Ethical Committee of Science for the Syddanmark region, Vejle, Denmark. All subjects were required to provide written, informed consent.

This study involved Chinese and Caucasian subjects undergoing elective surgery with propofol anesthesia, using NMB with rocuronium. For inclusion, subjects had to be 18–64 years of age and of American Society of Anesthesiologists Class 1–3. Chinese subjects had to be born in China, have never emigrated out of China and have a Chinese home address. Similarly, Caucasian subjects had to be born in Europe have never emigrated out of Europe and have a European home address.

Subjects were excluded from the study if they had anatomical malformations expected to lead to difficult tracheal intubation, neuromuscular disorders affecting NMB, significant renal/hepatic dysfunction (as determined by the investigator), (family) history of malignant hyperthermia, allergy to general anesthesia medications, contraindication to study drugs or a clinically significant condition that may interfere with the trial (as determined by the investigator).

Screening took place ≤ 7 days before study treatment administration. Eligible subjects were randomized via a central randomization system. The sponsor produced a computer-generated randomization schedule with treatment codes in blocks, using a validated SAS-based application. The schedule associated each treatment code with a subject number, and subjects were randomized in a 1:1 ratio to receive either sugammadex 2 mg/kg or neostigmine 50 μg/kg with atropine 10–20 μg/kg.

### Study procedures

An intravenous (IV) cannula was inserted for the administration of anesthetic drugs. Anesthesia was induced and maintained with IV propofol according to the clinical needs of the subject. Opioids could be administered according to local practice. Other anesthetic practices, e.g. use of methods such as bispectral index or entropy monitoring for measuring depth of anesthesia, were performed according to routine practices at the study site. Propofol was administered until a train-of-four (TOF) ratio of 0.9 has been established by the TOF-Watch® SX (Organon Ireland Ltd., a subsidiary of Merck and Co., Swords, Co. Dublin, Ireland).

After induction of anesthesia but before rocuronium administration, neuromuscular monitoring was carried out using continuous acceleromyography at the adductor pollicis muscle using the TOF-Watch® SX, in agreement with guidelines for Good Clinical Research Practice in pharmacodynamic studies of neuromuscular blocking agents [8,9]. Following induction of anesthesia, the TOF-Watch® SX was calibrated with the built-in calibration modus (CAL 2) after 5-sec 50 Hz tetanic stimulation preceded by a repetitive TOF stimulation for 1 min. After calibration, a 3–4-min repetitive TOF stimulation was required before administration of rocuronium to ensure a stable response. TOF stimulation was applied every 15 sec at the ulnar nerve until the end of anesthesia or at least until recovery of the TOF ratio to 0.9. Neuromuscular data were collected via an interface to a computer by means of the TOF-Watch® SX Monitoring Program, version 2.3.

After the TOF-Watch® SX had been set up, rocuronium 0.6 mg/kg was administered within 10 sec as a fast-running IV infusion. Tracheal intubation was then performed. Rocuronium maintenance doses of 0.1–0.2 mg/kg were given as required throughout anesthesia. After the last dose of rocuronium, at reappearance of second twitch, a single IV dose of sugammadex 2 mg/kg or a single IV dose of neostigmine 50 μg/kg plus atropine 10–20 μg/kg was administered to reverse NMB. All doses of neuromuscular blocking agents and reversal agents were administered based on actual body weight.

### Efficacy analyses

The primary efficacy variable was the time from the start of administration of sugammadex or neostigmine/atropine to recovery of the TOF ratio to 0.9. Secondary efficacy variables included time to recovery of the TOF ratio to 0.7 and 0.8.

In line with mean data from other studies within the sugammadex clinical trial programme, time to recovery of the TOF ratio to 0.9 > 6 min for a subject receiving sugammadex 2 mg/kg was classed as a prolonged recovery time.

### Safety

Safety assessments were performed by a safety assessor who was blinded to the treatment administered, and included monitoring of adverse events (AEs), vital signs and physical examination. Treatment-related AEs were those AEs considered by the investigator to be related to the study treatment (sugammadex or neostigmine). Risk factors for post-operative nausea and vomiting (PONV) [10] were assessed at baseline, and the relationship between the number of risk factors and occurrence of PONV during the study was evaluated. Additionally, subjects were assessed for evidence of residual NMB or recurrence of NMB, using both the TOF-Watch® SX and clinical signs.

In addition to safety assessments during screening and the peri-operative period, further assessments were made at a post-anesthetic visit (10 h after study drug administration, or the day following the operation) and at the follow-up assessment on Day 8.

### Statistical analysis

For regulatory considerations of a new pharmaceutical compound in China, 100 evaluable subjects per treatment group are required. For the primary objective, assuming a maximum drop-out rate of 13%, 115 evaluable subjects were to be enrolled per treatment group across the Chinese sites. It was estimated that this would give a power of at least 95% to demonstrate that recovery of the TOF ratio to 0.9 after sugammadex 2 mg/kg is at least two times faster than after neostigmine 50 μg/kg, testing at a significance level of 5% (two-sided), and assuming that the coefficient of variation (CV) in both groups is < 2.2 (based on previous sugammadex studies). For the secondary objectives, enrollment of 30 Caucasian subjects per treatment group was required to ensure a power of 80% to demonstrate equivalence in recovery times between Chinese and Caucasian subjects (assuming a standard deviation of 1.5 min), and a power of 95% to demonstrate at least three times faster recovery with sugammadex 2 mg/kg vs neostigmine 50 μg/kg in Caucasian subjects (with a CV of 1.1).

The primary analysis was performed on the full analysis set (equivalent to an intent-to-treat (ITT) population, which included all subjects who received the randomized study drug and had at least one efficacy assessment. In the event of missing data, values were imputed, using a worst-case scenario for sugammadex [7].

For the primary analysis, logarithms of the times from start of sugammadex and neostigmine administration to recovery of the TOF ratio to 0.9 in the Chinese subjects were compared using analysis of variance on log recovery times adjusted for study site effects. Recovery times are now known to follow an approximately lognormal distribution and thus recovery times were summarized by geometric means and associated 95% confidence Intervals (CIs).

Data for the secondary objective of demonstrating faster recovery in Caucasian subjects after sugammadex vs neostigmine were analyzed as for the primary objective. For the secondary objective of demonstrating equivalence in recovery times of the TOF ratio to 0.9 between Chinese and Caucasian subjects, analyses were performed using a non-parametric CI approach, which enables a quantitative measure of any differences between the groups. The estimated median difference between the two subject groups and the corresponding two-sided 97.5% CI were calculated using the Hodges–Lehmann estimator for treatment effect and Moses for CI of estimated treatment effect. Equivalence in efficacy between Chinese and Caucasian subjects was considered to be demonstrated when the 97.5% CI for the median difference between the groups was within a -1 to +1 min interval.

Safety analyses were performed for the all-subjects-treated (AST) group, and included all randomized subjects who received a dose of study medication.

## Results

**Figure 1:**
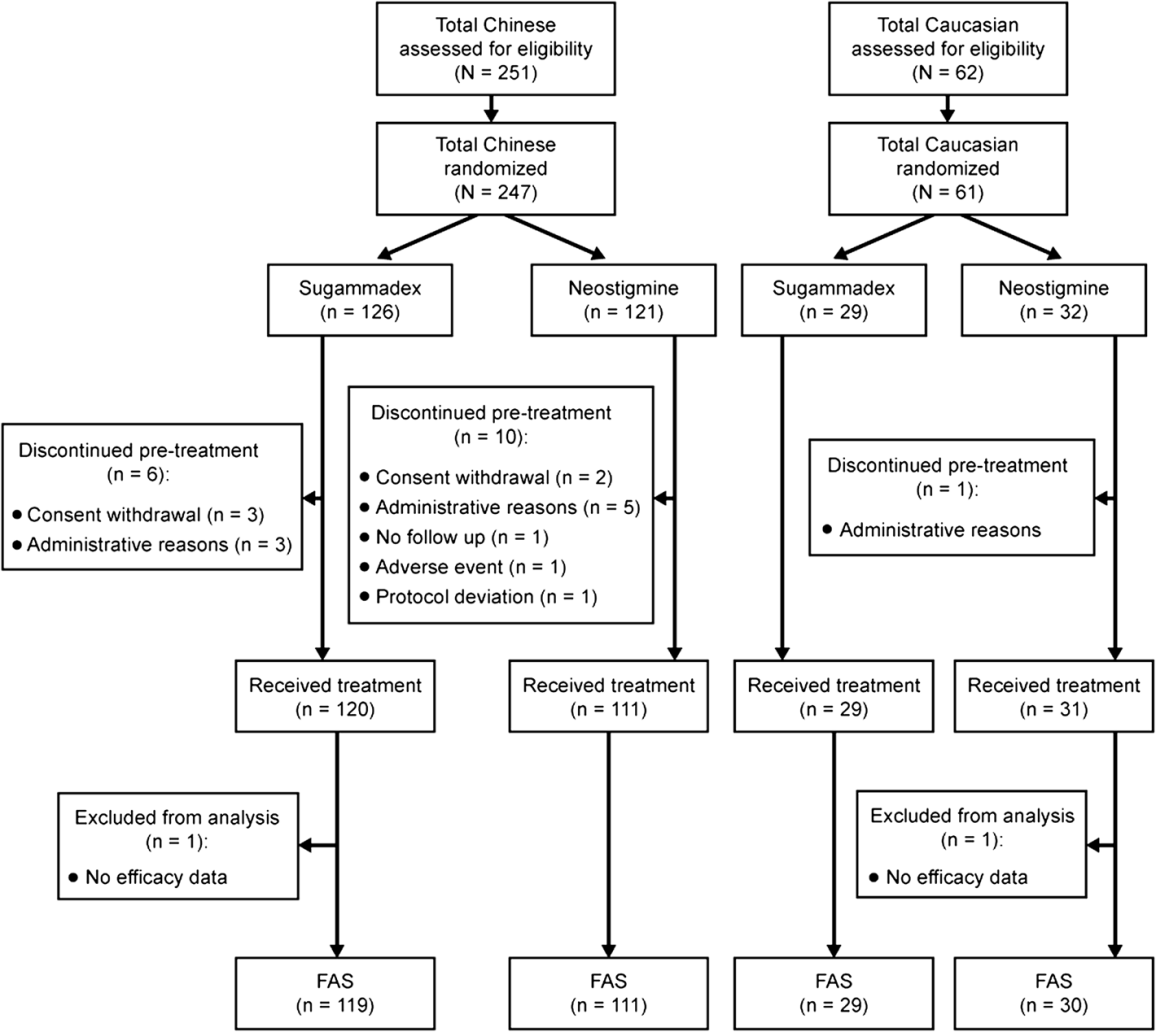
**Patient disposition and flow through the study (in accordance with CONSORT guidelines). ***FAS*: full analysis set.

Patient disposition and flow (according to CONSORT guidelines) is shown in Figure 1. Of 247 randomized Chinese subjects, 16 subjects discontinued the study, and one subject who completed the study had missing efficacy data. Hence, 231 Chinese subjects received study treatment and were included in the safety analysis (AST group) and 230 Chinese subjects with evaluable data were included in the efficacy analysis (full analysis set; sugammadex, n = 119, neostigmine, n = 111). In total, 61 Caucasian subjects were randomized, of whom 60 received treatment (AST group), and 59 had evaluable data (full analysis set; sugammadex, n = 29, neostigmine, n = 30) (Figure 1).

Baseline characteristics were comparable within subject groups between subjects receiving sugammadex and those receiving neostigmine (Table 1). Anesthesia was induced and maintained with propofol in all Chinese and Caucasian subjects. The most frequently administered opioid was remifentanil (Table 1).

**Table 1:** Baseline characteristics and anesthetic agents received (all-subjects-treated group)

	**Chinese**		**Caucasian**	
	**Sugammadex (n = 120)**	**Neostigmine (n = 111)**	**Sugammadex (n = 29)**	**Neostigmine (n = 31)**
Gender (n, %)				
Female	78 (65)	86 (77)	25 (86)	28 (90)
Male	42 (35)	25 (23)	4 (14)	3 (10)
Race (n, %)				
White	0 (0)	0 (0)	29 (100)	31 (100)
Asian	120 (100)	111 (100)	0 (0)	0 (0)
Age (Mean, SD years)	39.9 (10.8)	39.4 (10.8)	52.0 (10.3)	51.9 (7.3)
Weight (Mean, SD kg)	62.8 (12.6)	61.3 (11.3)	72.8 (10.8)	72.7 (11.8)
Height (Mean, SD cm)	163.8 (7.7)	163.7 (6.7)	169.1 (6.6)	167.9 (6.8)
BMI (mean, SD kg/m^2^)	23.3 (3.6)	22.8 (3.3)	25.5 (3.5)	25.9 (4.3)
ASA class (n, %)				
1	90 (75)	82 (74)	12 (41)	16 (52)
2	28 (23)	29 (26)	15 (52)	15 (48)
3	2 (2)	0 (0)	2 (7)	0 (0)
Anesthetic agents received (n, %)				
Propofol	120 (100)	111 (100)	29 (100)	31 (100)
Remifentanil	101 (84)	92 (83)	20 (69)	23 (74)
Fentanyl	65 (54)	66 (59)	7 (24)	10 (32)
Sufentanyl	41 (34)	38 (34)	9 (31)	8 (26)
Tramadol	2 (2)	3 (3)	0 (0)	0 (0)
Butorphanol	0 (0)	1 (1)	0 (0)	0 (0)

*ASA*: American Society of Anesthesiologists; *BMI*: Body mass index; *SD*: Standard deviation.

### Efficacy analyses

**Figure 2:**
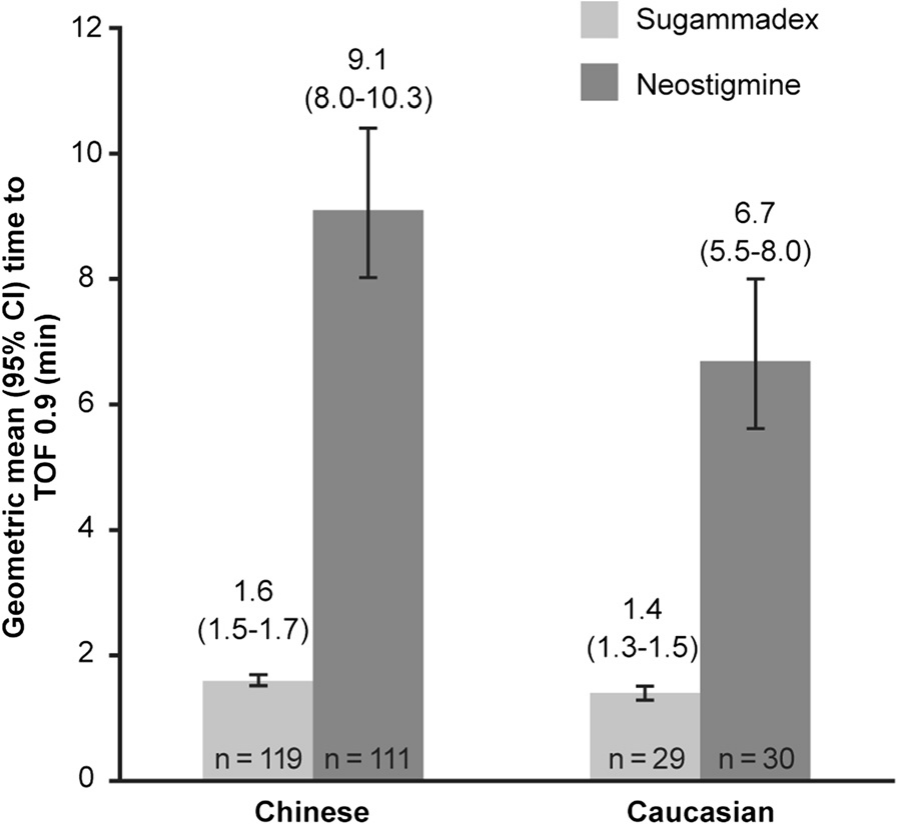
Time from start of administration of sugammadex or neostigmine to recovery of the train-of-four (TOF) ratio to 0.9 in Chinese and Caucasian subjects (full analysis set).

**Figure 3:**
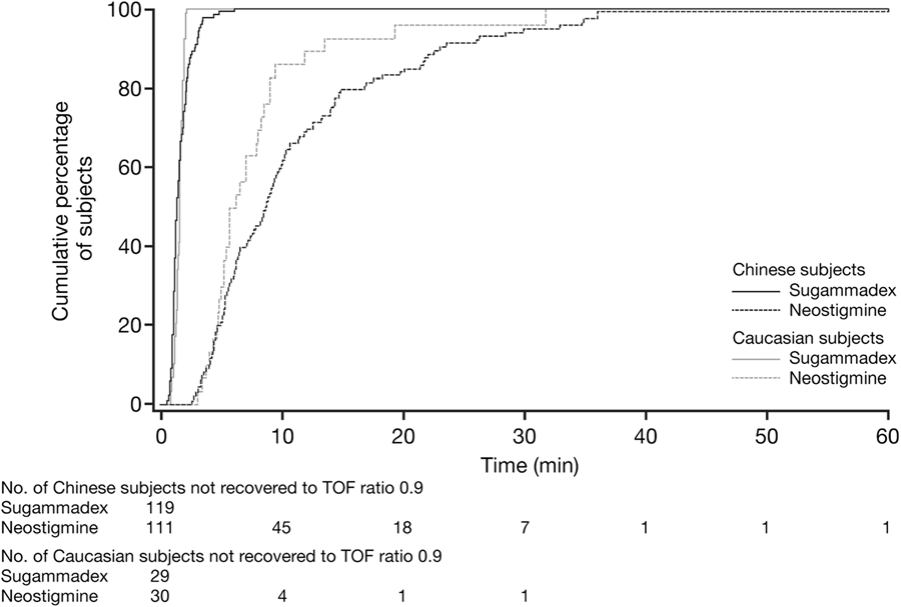
Cumulative percentage of Chinese and Caucasian subjects recovering to a train-of-four (TOF) ratio of 0.9 over time (full analysis set).

In the Chinese subjects, geometric mean (95% CI) time to recovery of the TOF ratio to 0.9 was 1.6 (1.5–1.7) min with sugammadex vs 9.1 (8.0–10.3) min with neostigmine (Figure 2). In total, 91% of Chinese subjects recovered to a TOF ratio of 0.9 within 3 min after administration of sugammadex, whereas only 1.8% recovered within 3 min after neostigmine. Fastest and slowest times to recovery following neostigmine were 2.7 and 60.4 min, respectively. Figure 3 shows the cumulative percentage of Chinese and Caucasian subjects recovering to a TOF ratio of 0.9 over time. Recovery time was estimated to be 5.7 times faster with sugammadex vs neostigmine in the Chinese subjects (95% CI 4.9–6.6; *p* < 0.0001).

An outlying recovery time of 6.2 min was observed for one Chinese subject after receiving sugammadex. For this subject, TOF 0.7 and 0.8 were reached at 0.9 min and 1.4 min after sugammadex, respectively, and were thus within normal ranges. However, due to technical issues with neuromuscular monitoring, an unusually high first twitch value affected the TOF ratio and caused the delay in reaching TOF 0.9.

In the Caucasian subjects, geometric mean (95% CI) time to recovery of the TOF ratio to 0.9 was 1.4 (1.3–1.5) min and 6.7 (5.5–8.0) min for those receiving sugammadex and neostigmine, respectively (Figure 2). All Caucasian subjects recovered to a TOF ratio of 0.9 within 2 min from start of administration of sugammadex, while none recovered within 2 min after receiving neostigmine. Fastest and slowest times to recovery following neostigmine were 3.0 and 31.4 min, respectively. In Caucasian subjects there was an estimated 4.8 times faster recovery with sugammadex vs neostigmine (95% CI 3.7–6.0; *p* < 0.0001).

Comparable geometric mean times to recovery to TOF 0.9 following treatment with sugammadex were observed in Chinese and Caucasian subjects (1.6 and 1.4 min, respectively; Table 2). The estimated median difference was 7 sec (97.5% CI -5, 21 sec). The 97.5% CI was within the pre-specified range of -1 to +1 min, supporting equivalence of efficacy between Chinese and Caucasian subjects. Following neostigmine treatment, recovery was 1.37 (95% CI: 1.05–1.78) times faster in Caucasian vs Chinese subjects (6.7 vs 9.1 min, respectively).

**Table 2:** Comparison of time (min) from administration of sugammadex vs neostigmine to recovery of the train-of-four (TOF) ratio to 0.7, 0.8 and 0.9 in Chinese and Caucasian subjects (full analysis set, with imputed data)

	**Chinese**		**Caucasian**	
	**Sugammadex (n = 119)**	**Neostigmine (n = 111)**	**Sugammadex (n = 29)**	**Neostigmine (n = 30)**
**TOF 0.7**				
Geometric mean (95% CI)	1.1 (1.1–1.2)	4.4 (4.0–4.9)	1.0 (0.9–1.1)	3.4 (3.0–3.8)
Range	0.4–2.1	1.7–32.4	0.6–1.7	1.9–7.9
**TOF 0.8**				
Geometric mean (95% CI)	1.3 (1.2–1.4)	6.0 (5.4–6.7)	1.2 (1.1–1.3)	4.6 (4.0–5.4)
Range	0.6–2.8	2.2–-59.6	0.8–1.7	2.4–14.1
TOF 0.9*				
Geometric mean (95% CI)	1.6 (1.5–1.7)	9.1 (8.0–10.3)	1.4 (1.3-1.5)	6.7 (5.5-8.0)
Range	0.6–6.2	2.7–60.4	0.8–2.0	3.0–31.4

*CI*: Confidence intervals.

*Two Chinese subjects (one sugammadex, one neostigmine) had missing times to recovery of the TOF ratio to 0.9. In addition, the Central Independent Adjudication Committee considered the time to recovery of the TOF ratio to 0.9 to be unknown in another six Chinese subjects (three sugammadex, three neostigmine). Values were therefore imputed for these eight subjects.

Faster recovery times with sugammadex vs neostigmine were also observed for TOF ratios of 0.7 and 0.8 in both Chinese and Caucasian subjects (Table 2). For time to recovery to TOF 0.7, estimated median difference (95% CI) between Chinese and Caucasian subjects was 5 sec (-2, 13 sec) and for time to recovery to TOF 0.8 it was 7 sec (-1, 14 sec).

No significant interaction between study site and treatment group was observed for the Chinese or European centers (*p* = 0.77 and *p* = 0.14, respectively). Similarly, no significant study site effect was observed (corresponding *p* values: *p* = 0.11 and *p* = 0.42).

### Safety

The most frequently reported AEs, regardless of relation to the study drug, were incision site pain, procedural pain, pyrexia, nausea and dizziness (Table 3). The percentage of subjects who experienced at least one post-treatment AE appeared somewhat lower with sugammadex vs neostigmine, and was similar between Chinese and Caucasian subjects: 70% and 69%, respectively, following sugammadex, and 82% and 84%, respectively, following neostigmine.

**Table 3:** Adverse events (AEs) occurring for ≥ 5% subjects in any treatment group, regardless of relation to study drug (all-subjects-treated group)

	**Chinese**		**Caucasian**	
	**Sugammadex (n = 120)**	**Neostigmine (n = 111)**	**Sugammadex (n = 29)**	**Neostigmine (n = 31)**
Subjects with any AE (n, %)	84 (70)	91 (82)	20 (69)	26 (84)
Abdominal pain	1 (1)	6 (5)	1 (3)	1 (3)
Upper abdominal pain	6 (5)	2 (2)	–	2 (6)
Nausea	10 (8)	13 (12)	4 (14)	7 (23)
Odynophagia	6 (5)	3 (3)	–	–
Vomiting	11 (9)	11 (10)	–	–
Fatigue	–	–	2 (7)	–
Pyrexia	16 (13)	16 (14)	2 (7)	1 (3)
Sensation of foreign body	7 (6)	4 (4)	–	–
Cardiac anesthetic complication	1 (1)	6 (5)	–	4 (13)
Incision site pain	28 (23)	26 (23)	–	–
Procedural hypotension	–	–	–	5 (16)
Procedural nausea	4 (3)	7 (6)	–	–
Procedural pain	10 (8)	10 (9)	13 (45)	12 (39)
Procedural vomiting	4 (3)	7 (6)	–	–
Wound complication	3 (3)	2 (2)	3 (10)	3 (10)
Dizziness	11 (9)	21 (19)	2 (7)	–
Headache	5 (4)	6 (5)	1 (3)	1 (3)
Insomnia	2 (2)	1 (1)	2 (7)	2 (6)
Vaginal hemorrhage	5 (4)	9 (8)	–	–
Increased upper airway secretion	5 (4)	10 (9)	–	–

AEs considered by the investigator to be possibly or probably treatment-related were reported for 9% and 3% of sugammadex-treated Chinese and Caucasian subjects, respectively, and for 18% and 35% of corresponding neostigmine-treated subjects. The most frequently reported treatment-related AE was anesthetic cardiac complication (in 11 subjects in total [1% and 0% of Chinese and Caucasian subjects after sugammadex and 5% and 13% of Chinese and Caucasian subjects after neostigmine, respectively]). All subjects with anesthetic cardiac complication were reported to have bradycardia or decreased heart rate. Other frequently reported treatment-related AEs were procedural hypotension (in five subjects in total [16% Caucasian subjects receiving neostigmine and no sugammadex-treated subjects]), and increased upper airway secretion (in six subjects in total [2% Chinese subjects receiving sugammadex and 4% Chinese subjects receiving neostigmine, and not reported in Caucasian subjects]). All AEs related to bradycardia or low heart rate were considered by the investigator to be possibly or probably treatment-related; this included all subjects with anesthetic cardiac complication as noted above, four subjects with procedural complication (three Chinese, one Caucasian, all in the neostigmine group), and one Chinese subject in the sugammadex group who had mild bradyarrhythmia. In total, two Chinese subjects (2%) and no Caucasian subjects in the sugammadex group and nine Chinese subjects (8%) and five Caucasian (16%) subjects in the neostigmine group had at least one bradycardia or low heart rate event.

Serious AEs were reported for three subjects, all following treatment with neostigmine, and all were considered unlikely to be related to the study drug by the investigator. One Chinese subject experienced incision site hemorrhage of severe intensity. Enterococcal bacteremia and anastomotic leak of moderate and severe intensity, respectively, were experienced by two Caucasian subjects.

Three subjects (one Chinese and two Caucasian) who received sugammadex and three subjects (all Chinese) who received neostigmine had AEs which were considered by the study sponsor to be potentially related to muscle weakness. Two of these AEs were considered by the investigator to be potentially treatment-related (mild hypoventilation in a Chinese subject who received sugammadex and severe muscular weakness in a Chinese subject who received neostigmine). Four of these AEs were considered unlikely to be treatment-related. The mild hypoventilation was treated with supplemental oxygen and resolved the next day; the subject had received three maintenance doses of rocuronium and a single IV dose of tramadol 100 mg at 3 min before sugammadex administration. The severe muscular weakness (weakness of lower limbs) began 7 h after neostigmine administration and resolved the next day; the subject experiencing this AE received no maintenance rocuronium dose and received a prescribed dose of gentamicin 80 000 U at 6 h after neostigmine administration. There was no evidence of residual NMB or recurrence of NMB, either clinically or based on neuromuscular monitoring for any patient.

Ten Chinese subjects (seven receiving sugammadex and three receiving neostigmine) had AEs considered by the study sponsor to be potentially attributable to drug hypersensitivity (e.g. mild-to-moderate rash, pruritis, facial swelling, facial flushing). All except one of these AEs (moderate rash on chest following sugammadex) were considered by the investigator to be unlikely to be related to the study drug. This subject, who experienced moderate rash on the chest, was treated with diphenhydramine and recovered on the same day. There were no reports consistent with anaphylaxis.

Events considered by the investigator to be likely reflective of bleeding were reported for eight (7%) Chinese subjects treated with sugammadex (five cases of vaginal hemorrhage, plus a case each of incision site hemorrhage, uterine hemorrhage and occult blood stool), and for 12 (11%) Chinese subjects treated with neostigmine (nine cases of vaginal hemorrhage, one case of incision site hemorrhage, and two cases of melaena). One (2%) Caucasian subject had a bleeding event; this patient experienced post-procedural hematoma after lumpectomy surgery, and was treated with neostigmine. None of these events were considered by the investigator to be related to study medication.

In total, PONV events were reported for 16 Chinese (13%) and four Caucasian (14%) subjects who were treated with sugammadex and 19 Chinese (17%) and seven Caucasian (23%) subjects who were treated with neostigmine. Each subject had at least one PONV risk factor with the majority of subjects having three risk factors.

## Discussion

This was the first study comparing efficacy and safety of sugammadex 2 mg/kg vs neostigmine 50 μg/kg plus atropine 10–20 μg/kg for reversal of moderate rocuronium-induced NMB in Chinese and Caucasian subjects. Neostigmine was chosen as the comparator reversal agent as it is commonly used for NMB reversal in many countries. Atropine was chosen as the anticholinergic agent to reflect current clinical practice in China.

The main study finding was that recovery to a TOF ratio of 0.9 was significantly faster after administration of sugammadex 2 mg/kg compared with neostigmine 50 μg/kg (geometric mean 1.6 min vs 9.1 min, respectively). Geometric mean time to a TOF ratio of 0.9 in Caucasian subjects was 1.4 min and 6.7 min for sugammadex and neostigmine, respectively, confirming the efficacy of sugammadex 2 mg/kg for reversal of moderate rocuronium NMB in Caucasian subjects as demonstrated in previous studies [3,11,12]. Furthermore, equivalence of efficacy of sugammadex (i.e. time to recovery of the TOF ratio to 0.9) was demonstrated between Chinese and Caucasian subjects.

The overall safety profile of sugammadex was shown to be favorable, and no serious AEs reported in sugammadex-treated subjects. The most frequently reported AEs were incision site pain, procedural pain and nausea. Importantly, there was no evidence of recurrence of NMB in any patient, either clinically or according to neuromuscular monitoring.

A higher number of subjects in the neostigmine group had at least one bradycardia or low heart rate event compared with those in the sugammadex group. Bradycardia is a side-effect associated with the use of acetylcholinesterase inhibitors such as neostigmine, although co-administration with an anticholinergic agent would be expected to counteract this [4]. It should be noted that, in the current study, neostigmine 50 μg/kg was given together with 10–20 μg/kg atropine in a ratio ranging from 2.5:1 to 5:1, depending on local practice.

A total of seven Chinese subjects who received sugammadex and three who received neostigmine had AEs consistent with drug hypersensitivity reactions, although only one of these was considered by the investigator to be possibly treatment-related (moderate rash on chest following sugammadex). None of the drug hypersensitivity reports were consistent with anaphylaxis, and there were no reports of drug hypersensitivity in Caucasian subjects. Low incidences of suspected sugammadex-induced hypersensitivity reactions have been reported in healthy volunteers [13,14]; Merck (Study P07042), data on file, and as post-marketing events in case-reports [15-17]. Reported reactions have varied from isolated skin reactions to serious systemic reactions (i.e. anaphylaxis, anaphylactic shock) and have occurred in patients with no prior exposure to sugammadex [18]. There have also been case reports of allergic drug reactions [19] and anaphylaxis [20] following neostigmine, although these reports are rare.

Overall, there were a total of 20 (9%) Chinese subjects and one (2%) Caucasian subject for whom, at least one event of bleeding was reported. The incidence of bleeding was higher in Chinese patients, regardless of treatment group, occurring in eight (7%) Chinese patients receiving sugammadex and 12 (11%) receiving neostigmine. Underlying reasons for this are not clear, although may be reflective of differences in surgical procedures. None of the events were considered treatment-related. Of note, while previous Phase I studies demonstrated a prolongation of activated partial thromboplastin time and prothrombin time (international normalized ratio) in healthy volunteers following sugammadex treatment [21,22], these effects were not clinically relevant, and sugammadex has since been shown not to be associated with an increased risk of bleeding vs usual care [23].

There also appeared to be some general differences between the populations in the safety findings, with 23% of Chinese subjects in both study groups reporting incision site pain but with no reports of this AE for the Caucasian subjects. Such differences may be reflective of the different surgical procedures undergone by the Chinese vs Caucasian subjects. Additionally, while the MedDRA system of coding was used to ensure consistency wherever possible, variations in local practices also may have contributed to observed differences in the incidence of AEs.

As ethnicity and geographic location may affect the effectiveness and duration of action of drugs, it is important to investigate sugammadex in a variety of patient populations. Differences in drug action between Chinese and Caucasian/white subjects have been previously reported for several drugs, [24-26] with at least some of these differences likely to be due to differences in drug metabolism, e.g. resulting from variations in cytochrome P450 enzymes [27,28]. Inter-ethnic differences in drug pharmacokinetics may also reflect differences in lipid stores and body stature [29]. Importantly, the results of the present study have excluded any clinically relevant differences in the effectiveness of sugammadex 2 mg/kg in Chinese compared with Caucasian subjects. However, geometric mean times to recovery to TOF 0.9 following neostigmine administration were 1.37 (95% CI 1.05–1.78) times longer in Chinese vs Caucasian subjects. This result is statistically significant; however, it is unclear why this difference occurred. While there is no known race effect for neostigmine, Chinese subjects have previously been shown to be more sensitive to effects of atropine vs Caucasian subjects [26], and this may have potentially played a role. Furthermore, spontaneous recovery from rocuronium has previously been shown to be somewhat slower in Chinese vs Caucasian patients [30], with the authors concluding that reasons for the observed interethnic difference were likely to be multifactorial.

In the current study, Chinese and Caucasian subjects treated with sugammadex had fewer anesthetic cardiac complications than those treated with neostigmine. All AEs termed “anesthetic cardiac complication” were incidences of bradycardia or low heart rate, a well-recognized potential side-effect of neostigmine [31].

In addition to the present study where sugammadex was administered at moderate blockade, a recent study demonstrated the efficacy and safety of sugammadex 4 mg/kg when administered at 1–2 post-tetanic counts (deep blockade) in Chinese patients [32], offering further support to the results of the current study.

## Conclusions

In Chinese subjects, sugammadex 2 mg/kg provided significantly more rapid reversal of moderate rocuronium-induced NMB compared with neostigmine (*p* < 0.0001). Efficacy of sugammadex 2 mg/kg was confirmed in Caucasian subjects, and equivalence of efficacy was demonstrated between Chinese and Caucasian subjects. Sugammadex 2 mg/kg was generally well-tolerated in both Chinese and Caucasian subjects.

## Abbreviations

AE: Adverse event; AST: All-subjects-treated; CI: Confidence interval; CV: Coefficient of variation; IV: Intravenous; NMB: Neuromuscular blockade; PONV: Post-operative nausea and vomiting; TOF: Train-of-four.

## Competing interests

HR is an employee of MSD, Oss, The Netherlands and TW is an employee of Merck Sharp & Dohme Corp., Whitehouse Station, NJ, USA, both of whom may own stock and/or hold stock options in the Company. EA was formerly an employee of MSD, Oss, The Netherlands. XW, SY, JL, BV, LX, CC, VD, YY, HO and YH work for institutions which received research funding from Merck Sharp & Dohme Corp., Whitehouse Station, NJ, USA. BV and VD have also received research funding from Merck & Co., Inc. for previous studies.

## Authors’ contributions

All authors are responsible for the work described in this paper. All authors were involved in at least one of the following: [conception, design, acquisition, analysis, statistical analysis, interpretation of data] and [drafting the manuscript and/or revising the manuscript for important intellectual content]. All authors provided final approval of the version to be published.

## Authors’ information

Esther Abels formerly of MSD, Oss, The Netherlands.

## Pre-publication history

The pre-publication history for this paper can be accessed here:

http://www.biomedcentral.com/1471-2253/14/53/prepub
